# Lamb Wave-Based Damage Localization and Quantification in Composites Using Probabilistic Imaging Algorithm and Statistical Method

**DOI:** 10.3390/s22134810

**Published:** 2022-06-25

**Authors:** Jiahui Guo, Xianping Zeng, Qijian Liu, Xinlin Qing

**Affiliations:** School of Aerospace Engineering, Xiamen University, Xiamen 361005, China; guojiahui@stu.xmu.edu.cn (J.G.); zengxp@stu.xmu.edu.cn (X.Z.); qijianliu@xmu.edu.cn (Q.L.)

**Keywords:** Lamb wave, matching pursuit decomposition algorithm, damage quantification, probabilistic imaging algorithm

## Abstract

Quantitatively and accurately monitoring the damage to composites is critical for estimating the remaining life of structures and determining whether maintenance is essential. This paper proposed an active sensing method for damage localization and quantification in composite plates. The probabilistic imaging algorithm and the statistical method were introduced to reduce the impact of composite anisotropy on the accuracy of damage detection. The matching pursuit decomposition (MPD) algorithm was utilized to extract the precise TOF for damage detection. The damage localization was realized by comprehensively evaluating the damage probability evaluation results of all sensing paths in the monitoring area. Meanwhile, the scattering source was recognized on the elliptical trajectory obtained through the TOF of each sensing path to estimate the damage size. Damage size was characterized by the Gaussian kernel probability density distribution of scattering sources. The algorithm was validated by through-thickness hole damages of various locations and sizes in composite plates. The experimental results demonstrated that the localization and quantification absolute error are within 11 mm and 2.2 mm, respectively, with a sensor spacing of 100 mm. The algorithm proposed in this paper can accurately locate and quantify damage in composite plate-like structures.

## 1. Introduction

Composite materials have attracted interest in the fields of aviation and aerospace due to their excellent specific strength and stiffness properties. Consequently, they progressively evolved into the primary structural material for the current generation of aircraft [1,2]. However, low-velocity impact or long-term loading can cause delamination, debonding, matrix cracking, and other structural damage faults [3]. Moreover, damage to composites is prone to increase, and will reduce structural bearing capacity and strength. If no damage is discovered and addressed appropriately in the initiation state, it can facilitate severe damage or even structural failure [4]. Hence, it is crucial to continuously monitor the damage status of composite structures to enhance structural safety [5].

Lamb waves are regarded as an effective and promising structural health monitoring (SHM) approach since they can travel over a long distance with low attenuation and high susceptibility to interference on the propagation path [6]. Recently, various imaging algorithms have been proposed for damage detection, including a time reversal-based imaging algorithm [7], a tomography imaging technique [8], a reconstruction algorithm for the probabilistic inspection of damage [9], and a delay-and-sum damage-imaging method [10]. Previously, Lamb wave-based SHM demonstrated generally promising results in composite structures for damage identification and localization. However, there is still a great demand for a reliable algorithm for damage quantification of such structures. The process is a challenge because composite structures are anisotropic, and the complex wave interactions complicate the Lamb wave propagation mechanism [11,12]. Hence, it is urgent to develop a method for damage quantification that is applicable to composite structures, thereby guaranteeing their safety [13].

Accordingly, several researchers employed the correlation between damage index and related damage size for quantification [14,15,16]. Pillarisetti and Talreja [17] proposed a set of stress wave factors as quantification descriptors taken from the power spectral density distribution of Lamb wave signals. Additionally, the stress wave factors were connected with the crack density in composite laminates. Torkamani et al. [18] introduced a time–domain damage index called “normalized correlation moment” to detect and access the delamination. Finite element and experimental data validated its excellent performance in the assessment of delamination in composites. He et al. [19] demonstrated a method for crack-size estimation focused on normalized amplitude and phase, which has been confirmed with data from coupon specimens and lap-joint components. Jin et al. [20] monitored the propagation of defect zones in curved composite structures by energy and signal difference coefficient. However, to gain higher accuracy, the methods reviewed above necessitate collecting a particular amount of data in various structure states. Moreover, the mathematical relationship between damage index and damage severity was only applicable to the structure for which the data were collected. The built model cannot be used directly when the structure is changed, which leads to poor portability. Thus, a universal quantification method that does not require the collection of significant volumes of data ahead of time is required for damage monitoring.

In addition to utilizing a specified damage index to quantify damage, scattering sources have been found as Lamb wave signals reflect from the damage boundary [21,22]. Moreover, the damage images for structures were reconstructed by scattering sources. The scattering sources were determined by the temporal information time of flight (TOF) of Lamb waves. The extraction of TOF from Lamb wave signals was frequently studied in both time domain and time–frequency domain perspectives [23,24]. Gorgin et al. [25] and Lu et al. [21] presented an approach in which the scattered waves occur from the boundary of damage sites for each sensing path, and a convex hull of scattering sources give the approximate shape and size of the damage. Hu et al. [26] established a theory for estimating the size of through-thickness circular holes by analyzing the reflection intensity of Lamb waves. However, the above research mainly focuses on the quantification of aluminum plates. Considering the scattering source as the damage boundary is more applicable to isotropic materials. In the propagation of Lamb waves, the anisotropy of composites causes different velocities in all directions. Therefore, it is difficult to precisely compute the propagation distance of Lamb waves from their propagation time. The scattering sources spread around the damage center rather than precisely situating at the border of the damage when utilizing the approximate propagation velocity.

Concerning the problems above, further study is required to accurately identify the level of damage in composite materials so that damage can be quantified with greater accuracy and universality. This paper proposed a TOF-based method for damage localization and quantification on composite plates. The uncertainty of damage monitoring caused by the composite material properties and the wave interaction when Lamb waves propagate in the structure are considered. A detection method for damage localization and quantification from the probabilistic perspective was introduced. An active sensor network with pitch–catch configurations was established to generate and capture Lamb wave signals. The matching pursuit decomposition (MPD) algorithm [27,28] was employed to extract TOF as a signal processing tool. The through-thickness holes in composite plate were located utilizing the probabilistic imaging algorithm. Meanwhile, the elliptical trajectory was obtained by calculating the TOF of each sensing path, with the point in the anticipated trajectory closest to the damage center being regarded as a scattering source. Finally, a statistical approach was utilized to process the probability density distribution of scattering sources. Damage size was characterized by the kernel probability density distribution of scattering sources. Experiments were performed on composite plates with multiple damage cases to validate the proposed technique.

The layout of this paper is as follows: Section 2 gives a detailed description of damage location identification and size quantification methodologies. Experimental specimen and signal acquisition device are introduced in Section 3. Section 4 illustrates the experimental results and gives the discussions. Finally, Section 5 summarizes the conclusions.

## 2. Damage Detection Method

**Figure 1:**
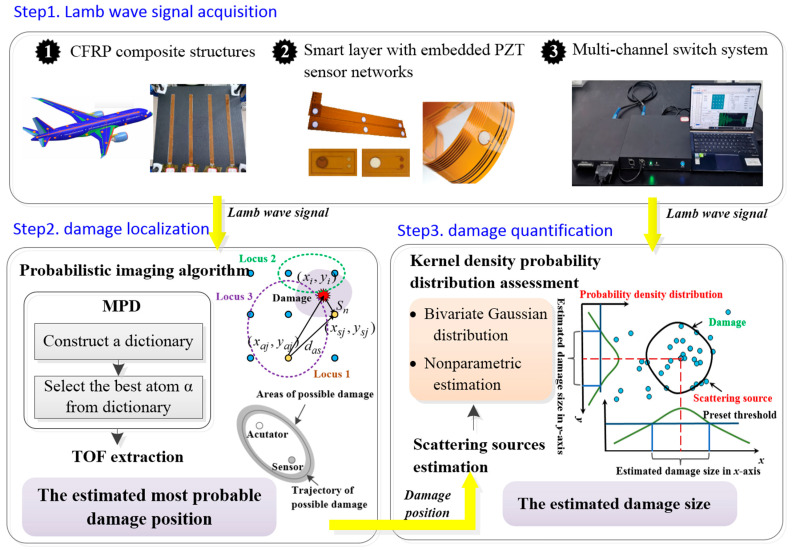
The flowchart of the experiment.

This paper proposes a damage detection method to locate and quantify the damage to a composite plate. The location of the damage is recognized by the TOF-based probability imaging algorithm. The accuracy of damage localization directly affects the quantitative analysis of the damage. On the basis of localization, the position of the corresponding scattering source is detected through each sensing path. The damage is quantified by analyzing the probability density distribution of scattering sources. The flowchart of the experimental process of this paper is shown in Figure 1.

### 2.1. Damage Localization Algorithm

#### 2.1.1. Probabilistic Imaging Algorithm by TOF

**Figure 2:**
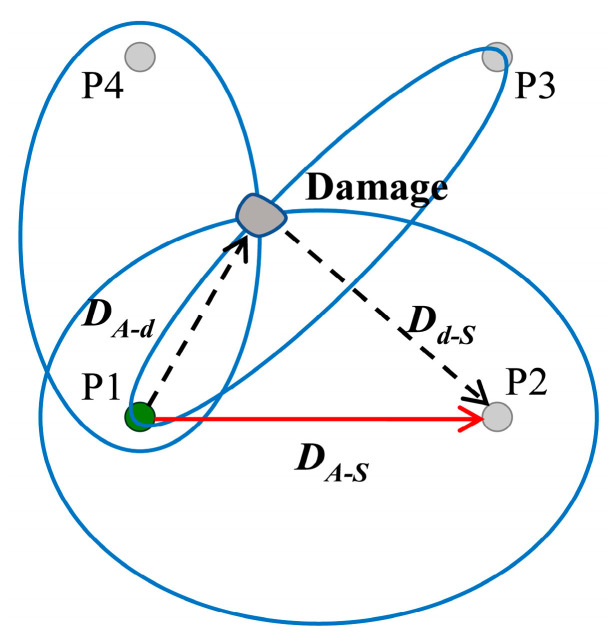
Ellipse positioning method.

**Figure 3:**
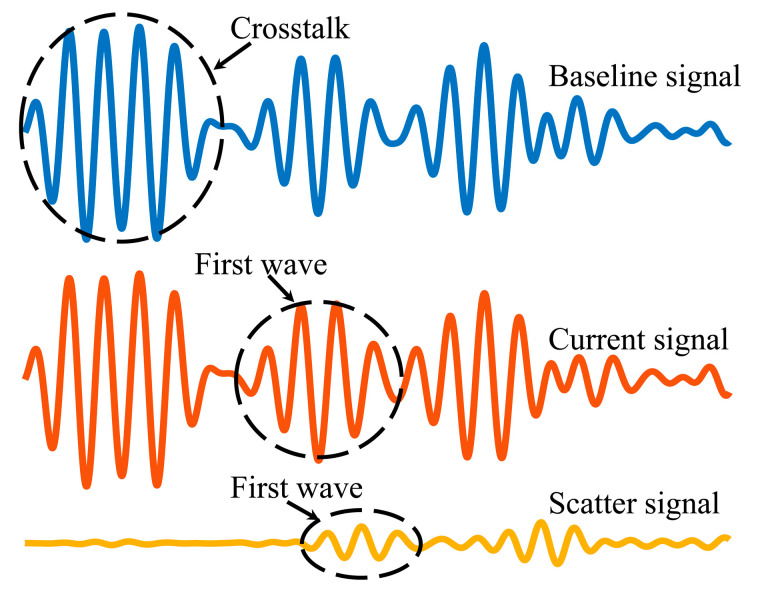
Lamb wave signals.

Lamb waves propagate after being activated in the structure, and can be detected by sensors arranged in various locations [29]. The damage location can be determined by the ellipse-positioning method once the TOF of Lamb waves in the undamaged and damaged structures of each sensing path have been acquired [30,31]. The monitoring network composed of four sensors is implemented as shown in Figure 2. The piezoelectric transducer (PZT) serves as both the actuator and the sensor. “P1” is the actuator, which excites a five-cycle sinusoidal signal modulated by the Hanning window, and “P2”, “P3”, and “P4” are the sensors used to collect Lamb wave signals excited by the actuator. Figure 3 shows the detected Lamb wave signals. The detected Lamb wave signals are utilized as a baseline signal through a direct path $D_{A-S}$ between the specified sensing path without structural damage. Hence, the arrival time of the first-wave packet is analyzed as the time required for Lamb waves passing through path $D_{A-S}$, denoted as ${TOF}_{B}$. Similarly, Lamb waves transmit from the actuator to the damage $D_{A-d}$ and scatter from the damage to the sensor $D_{d-S}$ when the structure is damaged. The difference between the baseline and current signals is utilized to obtain the scattered waves. During this process, the arrival time of scattered waves is recorded as ${TOF}_{S}$.

As shown in Figure 2, an elliptical trajectory with the actuator and the sensor as focal points can be obtained by satisfying the solution defined as:

(1) $$\frac{D_{A-d}+D_{d-S}}{D_{A-S}}=\frac{\sqrt{{(\;x_{A}-x_{d}\;)}^{2}+{(\;y_{A}{-y}_{d}\;)}^{2}}+\sqrt{{(\;x_{S}-x_{d}\;)}^{2}+{(\;y_{S}-y_{d}\;)}^{2}}}{\sqrt{{\left(\;x_{A}-x_{S\;}\right)}^{2}+{(\;y_{A}-y_{S}\;)}^{2}}}=\frac{{TOF}_{S}v_{gS}}{{TOF}_{B}v_{gB}}=\beta ,$$

where $v_{gB}$, $v_{gS}$ are the group velocities, $x_{A\;}$, $y_{A\;}$ are the coordinates of the actuator, $x_{S}$, $y_{S}$ are the coordinates of the sensor, and $x_{d}$, $y_{d}$ are the coordinates of the damage center. Only one ellipse is insufficient to pinpoint the site of the damage, and damage location can be accomplished by getting more than two ellipses from the sensor network utilizing different sensing paths. Since the ratio of ${TOF}_{B}$ and ${TOF}_{S}$ is fixed, the ratio *β* can be used to calculate the path length of scattered waves.

**Figure 4:**
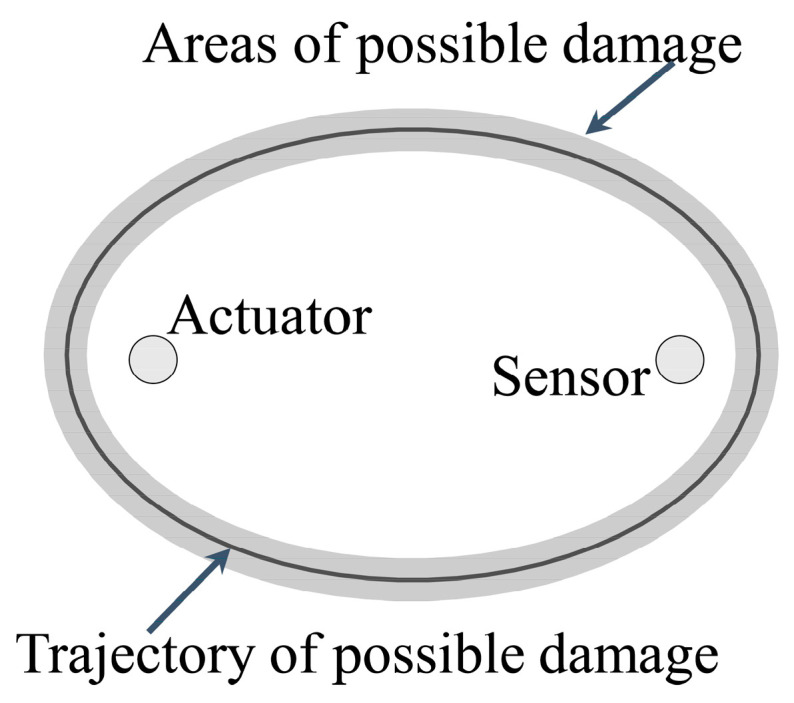
Probability imaging algorithm.

Because of the anisotropy of composites, it is difficult to correctly compute the propagation velocity in all directions. This work made an approximation to increase the algorithmic feasibility and efficacy. When propagating on both direct path and scattering path, it is considered that the Lamb waves have the same average velocity. However, such an approximation results in an incorrect distribution of outcomes outside the actual damage boundary. Accordingly, the probability imaging algorithm is proposed based on the ellipse positioning method as Figure 4 shows. In addition, a Gaussian distribution is employed to enhance the trajectory coverage area and decrease the localization error. Furthermore, the probability imaging algorithm grids the monitoring area of the sensor network into uniform pixel points [32]. The damage probability of each pixel point in the elliptical influence area of each sensing path is superimposed [31]. Overall, the damage to the structure can be intuitively portrayed by computing the damage probability of each pixel point on the structure.

Lamb wave signals are collected by specified sensing path before and after damage, and the ratio *β* in Equation (1) is determined after signal processing. The damage probability is estimated by computing the relative distance between each pixel in the monitoring area of each sensing path. In addition, the size of each pixel is 0.1 mm × 0.1 mm in this paper. The relative distance of each pixel (*x*, *y*) is calculated as follows:

(2) $$R_{j}\left(x,\;y\right)=\frac{\sqrt{{(\;x_{A}-x\;)}^{2}+{(\;y_{A}-y\;)}^{2}}+\sqrt{{(\;x_{S}-x\;)}^{2}+{(\;y_{S}-y)}^{2}}}{\sqrt{{\left(\;x_{A}-x_{S\;}\right)}^{2}+{(\;y_{A}-y_{S}\;)}^{2}}},$$

where $R_{j}(x,\;y)\;$is the relative distance of the pixel point (*x*, *y*) using the *j*th sensing path. The damage probability of the pixel point *f* (*x*, *y*) is determined by the normal distribution function which is written as follows [31]:

(3) $$f_{j}\left(x,\;y\right)=\frac{1}{\sigma \sqrt{2\pi }}e^{-\frac{{{(R}_{j}\left(x,\;y)-\mu \right)}^{2}}{{2\sigma }^{2}}},$$

(4) $$R_{j}\left(x,\;y\right)=\frac{D_{A,\;j-d}+D_{d-S,\;j}}{D_{A,\;j-S,\;j}}=\frac{{TOF}_{S}}{{TOF}_{B}}=\beta_{j},$$

$D_{A,\;j-S,\;j}$ is the distance between the actuator and the sensor of the *j*th path, $D_{A,\;j-d}$ and $D_{d-S,\;j}$ are the distances of the pixel (*x*, *y*) from the actuator to the sensor of the *j*th path, respectively.

**Figure 5:**
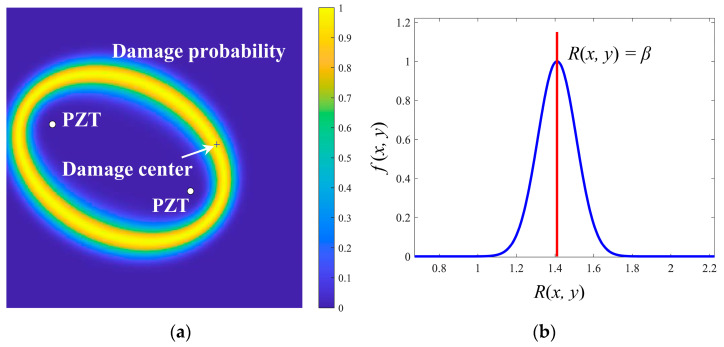
Damage probability distribution of specific paths. (**a**) Damage probability distribution; (**b**) The normal distribution.

As shown in Figure 5b, the coordinates of the pixel points (*x*, *y*) have the highest damage probability when the pixel fulfills Equation (4). The damage probability results in the monitoring region are calculated by a specific path as illustrated in Figure 5a, where “+” represents the actual damage center and the white circles represent the sensors. The damage probability value of each pixel point (*x*, *y*) in the monitoring area varies with relative distance $R_{j}\left(x,\;y\right)$. As the difference between $R_{j}(x,\;y)\;$and *β* increases, the probability of damage decreases.

(5) $$P(x\text{,}y)=\sum_{j=1}^{N}\;f_{j}\left(x,\;y\right),$$

Finding the pixel (*x*, *y*) with the highest damage probability value allows for damage localization. Moreover, if the monitoring region has *N* sensing paths in the monitoring area, the probability of damage at location (*x*, *y*) is written as Equation (5).

#### 2.1.2. TOF Extraction Method

The MPD algorithm is an effective signal processing tool to decompose the overlapped wave packets of a signal so that each wave mode can be identified. It is an iterative algorithm, which provides an accurate solution of signal decomposition based on the linear combination of unit basis functions composed of redundant dictionaries. In this paper, the MPD algorithm was utilized to reconstruct the Lamb wave signals and the direct wave packet of Lamb waves was extracted and its TOF was obtained. The MPD algorithm exploits the linear combination of unit basis functions or atomic functions in the dictionary *D* to express a signal accurately. This process is obtained by continuous iterative optimization by the following expressions:

*D* = [*d*_1_, *d*_2_ …… *d_i_*], (6)

(7) $$S=\sum_{k}\alpha_{k}d_{k},$$

where *d_i_* is the *i*th atom in *D*, *S* is the reconstructed signal linearly expressed by atoms in *D*. The MPD algorithm must choose the optimal collection of atoms representing the investigated signal from the overcomplete dictionary. Moreover, the ability of atoms to convey a signal is influenced by the qualities of basis vectors, which must match the original signal [33]. As a result, the MPD algorithm iterates through the most relevant set of atoms in a given dictionary to create a sparse representation of the signal. The MPD algorithm is broken down into the following phases.

(1) Construct a dictionary *D*

A dictionary is the normalizing basic module that defines the expression signal space. Dictionaries are comprehensive and redundant in most MPD algorithm applications. In this paper, the Hanning atomic set was selected [33], and the atomic expression is written as follows:

(8) $$\omega (t)=\frac{1}{2}\left[1-\cos\left(\frac{{2\pi f}_{c}\left(t-\tau \right)}{N}\right)\right],$$

(9) $$g(t)=\frac{1}{2}\left[1-\cos\left(\frac{2\pi f_{c}\left(t-\tau \right)}{N}\right)\right]\left[\sin\left({2\pi f}_{c}\left(t-\tau \right)\right)\right],$$

where *ω*(*t*) is the Hanning window function, *N* is the number of signal cycles, *g*(*t*) is the Hanning atom, $f_{c}$ is the center frequency of the analyzed signal, and *τ* is the translation factor.

(2) Select the best atom *α* from dictionary *D*

Initializing the signal residual $R^{0}$ = *s* (*s* is the input signal) and applying Equation (10) to obtain signal residual $R^{1}$, the selected atom $\alpha_{1}$ must meet Equation (11).

(10) $$R^{1}=s-<s,\alpha_{1}>\alpha_{1},$$

(11) $$\alpha_{1}=\arg_{d_{i}\in D}\max\left|<{s,d}_{i}>\right|.\;$$

Continue to decompose the residual $R^{1}$, Equation (12) is employed to extract the signal residual $R^{m}$ of each iteration. Meanwhile, creating a sparse approximation using the inner product of each atom in *D* and iterating until the accuracy is attained or the number of iterations is reached. It is critical to check that the chosen atom $\alpha_{m}$ matches the signal to the maximum extent feasible each time the analytical signal is estimated, as Equation (13) shows.

(12) $$R^{m}=R^{m-1}-<R^{m-1}{,\alpha }_{m}>\alpha_{m},$$

(13) $$\alpha_{m\;}=\arg_{d_{i}\in D}\max\left|<R^{m}{,d}_{i}>\right|.\;$$

Then, after *k* iterations, the reconstructed signal is expressed as follows:

(14) $$s_{re\;}=\sum_{m=1}^{k}<R^{m-1}{,\alpha }_{m}>\alpha_{m}.\;$$

The serial number of each wave component in the Lamb wave signals can be acquired after this signal decomposition method to characterize the TOF. The translation factor is utilized to construct the atoms during the atom set construction. Moreover, the translation factor grows sequentially, meaning that the atoms are translated in the index order. Recording the maximum projection length to get the most appropriate atom index. Hence, the atomic index can be applied to represent the TOF, which is introduced as follows:

(15) $$TOF=\frac{I}{S}\;,\;$$

where *I* is the calculated atomic index and *S* represents the sampling rate of the device.

### 2.2. Kernel Density Probability Distribution Assessment of Damage Size

**Figure 6:**
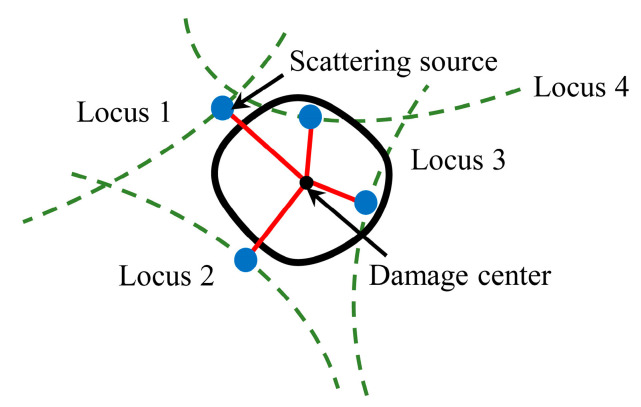
Scattering sources estimation.

The damage elliptical trajectory of each sensing path can be derived utilizing the ellipse positioning method. At the same time, the localization algorithm introduced in Section 2 can obtain the damage center. The position of the scattering source is determined by the shortest distance from the damage center on the trajectory of probable damage positions given by each sensing path as Figure 6 shows. Furthermore, a scatter plot of estimated scattering sources can be obtained by utilizing all monitoring paths in the sensor network.

However, the Lamb wave signals generated by the sensor network are frequently combined with a specific interference signal in the actual application process because of noise interference or changes in external environmental conditions. Moreover, the assumption that the average propagation velocity is a constant on both direct path and scattering path disabled scattering sources from being detected on the damage boundary. The scattering sources are densely dispersed around the damage center rather than evenly distributed on the damage boundary. Thus, statistical methods are utilized to determine the veracity of the data because there are a relative majority of preset sensors and sensing paths.

Kernel density estimation is a nonparametric method for estimating probability density function, which is applied in probability theory to estimate unknown density functions [34,35]. For instance, *x*_1_*, x*_2_ *... x_n_* are *n* samples of the independent distribution *F*, the probability density distribution function of *F* is described as *f*, and the kernel density estimation equation is written as follows:

(16) $$f_{h}\left(x)=\frac{\;1}{n}\sum_{i=1}^{n}\;K_{h}{(x-\;x}_{i}\right)=\frac{\;1}{nh}\sum_{i=1}^{n}K(\frac{x-x_{i}}{h}),$$

(17) $$K_{h}\left(x\right)=\frac{\;1}{h}K(\frac{x\;}{h}),$$

where *K* is the kernel function, which is a weight function. The kernel function controls the number of data utilized when estimating the value of $f_{h}$(*x*) at point *x*. $K_{h}\left(x\right)$ is the scaling kernel function and *h* is the standard deviation. In this paper, the Gaussian kernel function is selected to smooth the probability density distribution findings. The Gaussian kernel function is then given by the expression:

(18) $$K(x\;;\;h)\propto \;\exp\;(-\frac{x{\;}^{2}}{{2h}^{2}}).$$

**Figure 7:**
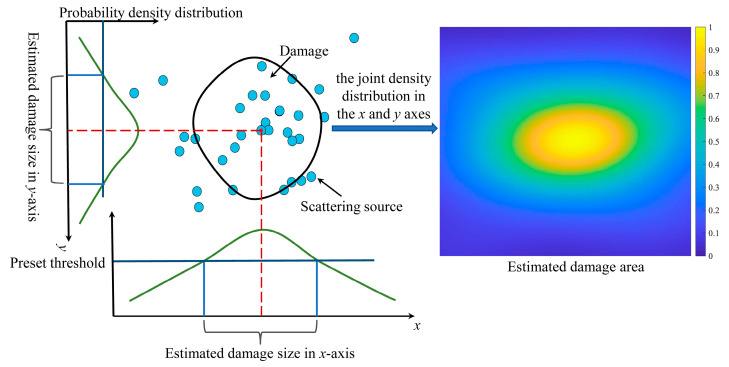
Damage area estimation method.

In the process of density estimation, a Gaussian kernel distribution is constructed for each sample. The kernel density estimation result is obtained by aggregating the final curves for the kernel function of each sample. Furthermore, the scattering sources distribution on the specimen required to be represented in two dimensions. The joint density distribution in the *x* and *y* axes was obtained to characterize the damage magnitude. Moreover, the size of the damage is calculated utilizing the probability density distribution of scattering sources, as Figure 7 shows. The damage can be described by showing the probability density distribution of the scattering sources on *x* and *y* axes. The probability density distribution of scattering sources in each axis direction is a one-dimensional curve, as shown in Figure 7. The two-dimensional distribution map of scattering sources can be created by combining the probability density distribution in *x* and *y* axis directions. By selecting a threshold, the probability distribution map can be digitized, and the pixels with a probability density larger than the threshold are considered as the genuine damage distribution, from which the numerical value of the damage size can be calculated. Typically, the threshold is chosen based on experimental data or experience.

## 3. Experiment

This research investigated a localization and quantification method for detecting through-thickness hole damage. Firstly, the damage was located utilizing the TOF-based probabilistic imaging algorithm. Simultaneously, the scattering source was recognized on the elliptical trajectory obtained through the TOF of each sensing path. Finally, damage size was characterized by the Gaussian kernel probability density distribution of scattering sources. In this regard, a CFRP composite plate was chosen as a specimen to validate the proposed method. The algorithm was validated by through-thickness hole damages in composites plate of various locations and sizes.

### 3.1. Specimen

**Figure 8:**
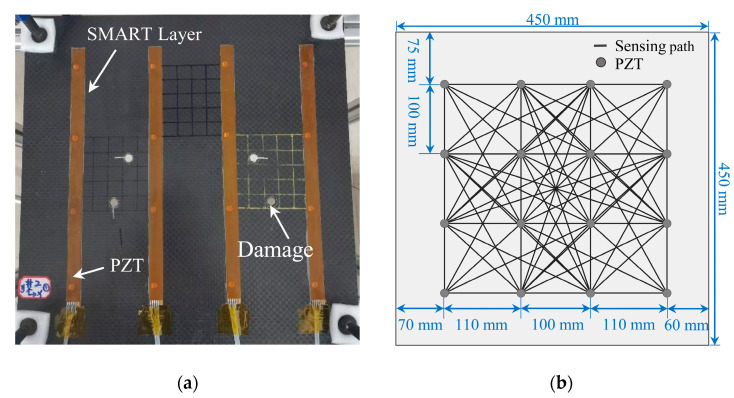
Experimental devices and sensor network layout. (**a**) Composite material plate-like specimen; (**b**) Preset sensor network.

The composite material plate-like specimen was 450 mm × 450 mm × 3 mm, as Figure 8a shows. The plate was constructed of six layers of carbon fiber woven cloth utilizing T700 12K with a quasi-isotropic layup of [0°/90°]_3_. Through-thickness holes with diameters of 4 mm, 8 mm, 12 mm were manufactured at the coordinates (127 mm, 185 mm) and (320 mm, 245 mm), taking the lower-left corner as the coordinate origin. The sensor network composed of 16 PZT was packaged in the SMART Layer [36] and mounted on the structure. The SMART Layer was made up of an embedded distributed piezoelectric sensor network that serves as both sensors and actuators for active and passive sensing in real-time. Meanwhile, four piezoelectric sensors were positioned in a square on the composite plate, and the composite material could be subdivided into nine distinct sections. The designed sensor network is depicted in Figure 8b, and there were 78 sensing paths in the monitoring area.

The integrity plate-like specimen was utilized to collect the Lamb wave signals on each sensing path as the baseline signal. Through-thickness holes in different locations with different diameters were drilled into the specimen artificially. The diameter of the hole was enlarged gradually from 4 mm to 12 mm in 4 mm increments at one position, and the sensor network was employed to collect signal after each manufacturing damage. Moreover, damage scattered signals could be calculated by computing the difference between the damage signal and baseline signal which could be applied to the subsequent damage localization and quantification.

### 3.2. Experimental Setup

**Figure 9:**
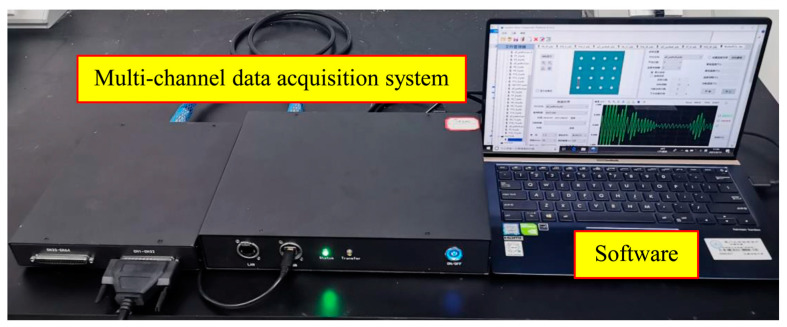
Data acquisition devices.

Lamb waves were generated and recorded utilizing a multi-channel data acquisition system built by the authors’ group, as shown in Figure 9. The multi-channel data acquisition system with a size of 223 mm × 201 mm × 49 mm and a host system with a size of 300 mm × 226 mm × 50 mm. In addition, the host was assembled with the waveform generator, voltage and charge amplifier, and the software. Integrated SHM software based on the Matlab software platform was developed to realize signal acquisition. The main technical indicators of the equipment are shown in Table 1.

The five-cycle tone burst signals modulated by the Hanning window were generated as an excitation signal at a central frequency of 60 kHz and recorded signals at a sampling rate of 12 MHz.

## 4. Results and Discussions

### 4.1. Damage Location Identification

The TOF of baseline signals and scattered signals detected on each sensing path was obtained by the MPD algorithm introduced in Section 2. The atomic index represented the TOF of Lamb waves through such a signal processing of extracting the direct wave to obtain the ratio *β*. Concurrently, the elliptical probability imaging algorithm based on TOF was employed to determine the location of damages with different diameters located at the coordinates (127 mm, 185 mm) and (320 mm, 245 mm).

**Figure 10:**
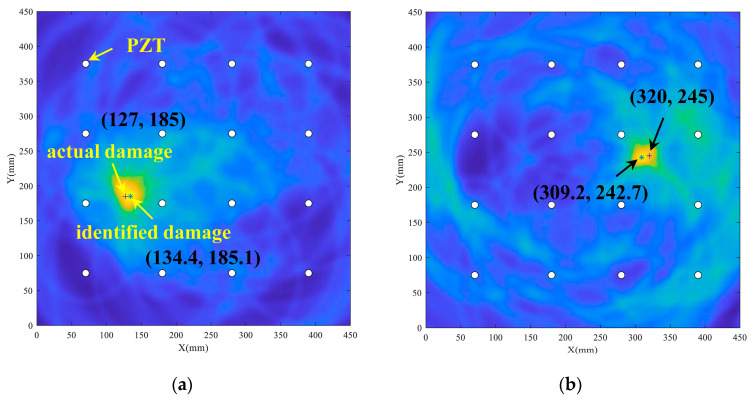

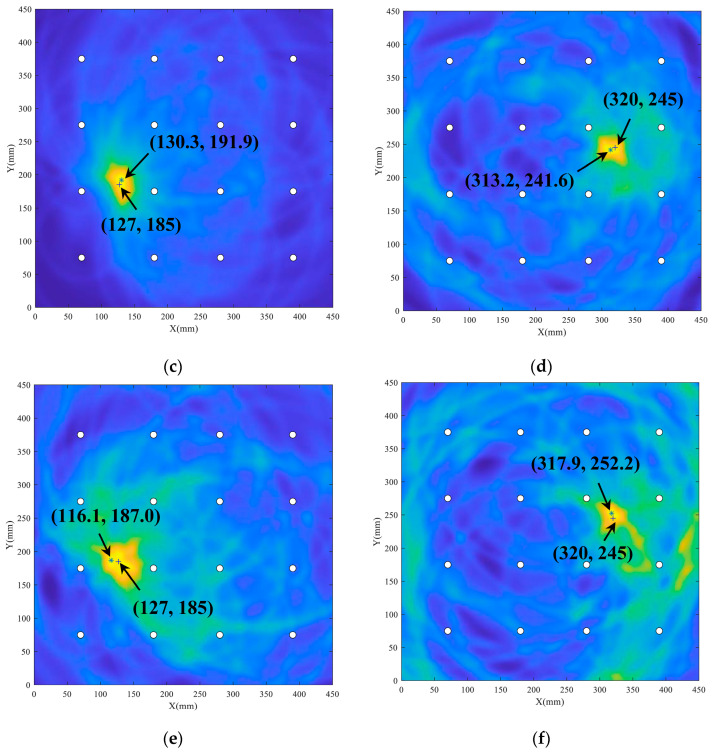
Damage localization results at different locations and sizes. (**a**) damage center (127, 185) and *d* = 4 mm; (**b**) damage center (320, 245) and *d* = 4 mm; (**c**) damage center (127, 185) and *d* = 8 mm; (**d**) damage center (320, 245) and *d* = 8 mm; (**e**) damage center (127, 185) and *d* = 12 mm; (**f**) damage center (320, 245) and *d* = 12 mm.

The results of the damage localization are given in Figure 10. The damage was enlarged gradually in steps of 4 mm in diameter at one position. Figure 10a,c,e are the results of the damage localization method with the center position (127 mm, 185 mm), and Figure 10b,d,f are the results of the damage localization method with the center position (320 mm, 245 mm). The “+” is the actual damage center position, and the “*” is the predicted damage center. From Figure 10, it is manifested that the probabilistic imaging localization results obtained by extracting the TOF using the MPD method are close to the actual damage center.

The damage center prediction findings and errors of the proposed method are detailed in Table 2. Accordingly, the experimental results revealed that the difference between the predicted and real damage locations was minimal with a relative error of 1.64%. Damage localization results indicated that the localization algorithm utilized in this study was capable of correctly determining the damage position. The probabilistic imaging localization results obtained by extraction of the TOF using the MPD method are more accurate than those obtained by extraction of the TOF using the Hilbert transform [37]. Meanwhile, the predicted location obtained by the two methods mentioned above was very close to the actual damage location. The results indicate that the TOF-based probabilistic imaging localization algorithm demonstrated in this paper is robust and accurate.

### 4.2. Damage Quantification

**Figure 11:**
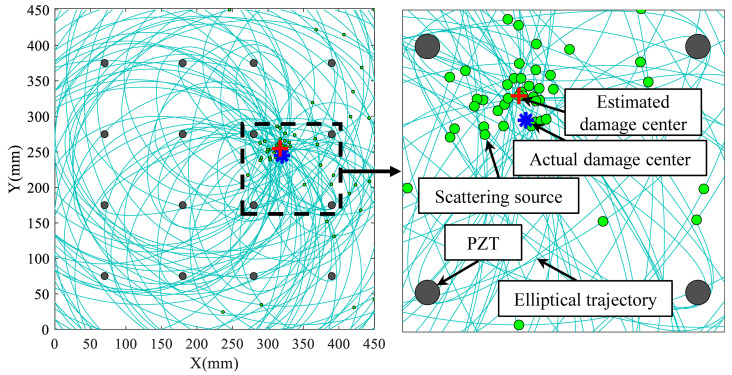
Distribution of scattering sources on the elliptical trajectory.

The TOF received from each sensing path can be applied to build an elliptical trajectory with the actuator and the sensor as the focal points. The point on the elliptical trajectory closest to the anticipated damage center was considered as the scattering source. A scatter distribution map of scattering sources was obtained by calculating the scattering sources on all sensing paths. Moreover, in the preconfigured sensor network, four piezoelectric sensors were positioned in a square to create a monitoring sub-area. The sub-intervals investigated can be chosen based on previously acquired damage localization results. Figure 11 depicts the scattering sources distribution produced by the sensor network under a specific damage state. Apparently, the scattering sources were majorly dispersed around the estimated damage center.

After the scattering sources were acquired, preliminarily processing the obtained scattering sources distribution utilizing the sub-interval. This process filtered out the scattering sources outside the sub-interval. The overlapping of scattering sources in Figure 11 reduced the number of scatter points. Therefore, the probability distribution was required to graphically portray the scattering sources distribution.

**Figure 12:**
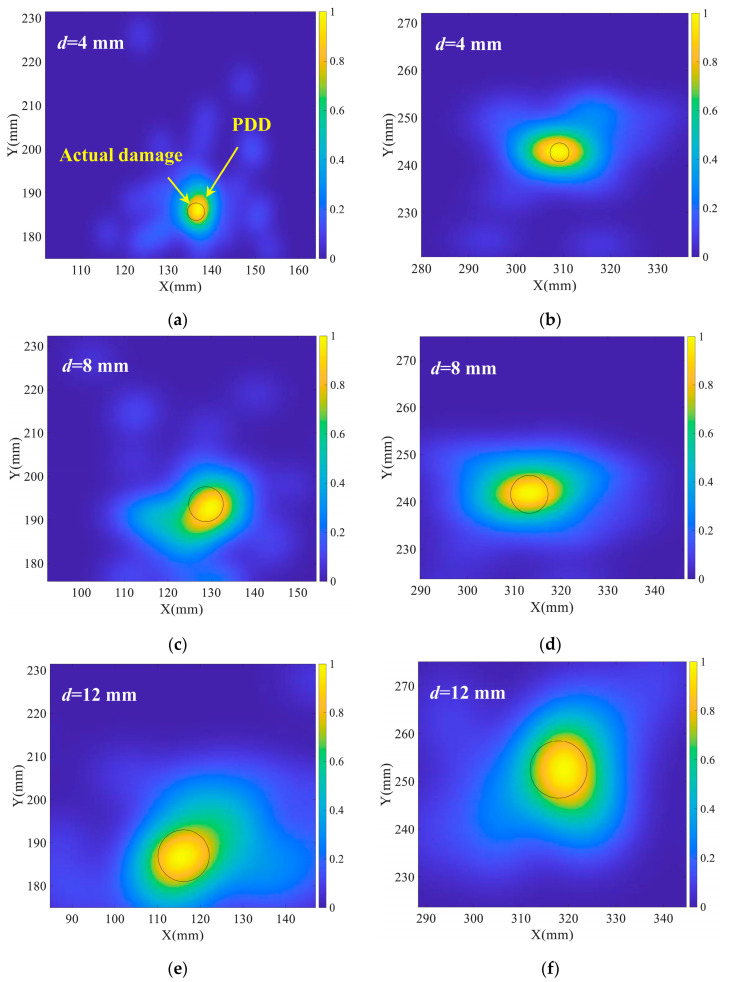
Quantification results of damage size. (**a**) damage center (127, 185) and *d* = 4 mm; (**b**) damage center (320, 245) and *d* = 4 mm; (**c**) damage center (127, 185) and *d* = 8 mm; (**d**) damage center (320, 245) and *d* = 8 mm; (**e**) damage center (127, 185) and *d* = 12 mm; (**f**) damage center (320, 245) and *d* = 12 mm.

The Gaussian kernel probability density distribution (PDD) function described the scattering sources distribution. Figure 12 shows the quantification results of damage with different sizes, which were enlarged gradually in steps of 4 mm in diameter at one position. Figure 12a,c,e shows the quantification results of damage with the center position (127 mm, 185 mm) and Figure 12b,d,f shows the quantification results of damage with the center position (320 mm, 245 mm). Meanwhile, the normalized Gaussian kernel probability density distribution results were presented in Figure 12, and the black circle represents the actual damage size in the predicted damage center.

As Figure 12 displays, the probabilistic imaging region appears to enlarge as the damage expands. The final quantification result of the damage was obtained by calculating the area with a normalized probability density greater than 0.8. Table 3 shows the damage size forecast results and errors. The estimation error of damage area was within 28 mm^2^. According to the estimated damage area, the corresponding diameter was determined, since the damage is round. Four of the six test points had a diameter estimation error of less than 1 mm, and only one had the error greater than 2 mm, which was 2.2 mm, indicating that the damage quantification method is extremely precise. The probability density distribution findings match the size of the actual damage. Simultaneously, damage expansion is described by the proposed damage quantification approach.

## 5. Conclusions

In this paper, an active Lamb wave-based damage detection algorithm utilizing MPD and the statistical method were proposed to locate and quantify damage. The algorithm was validated by through-thickness holes in composites plates of various locations and sizes. Based on the analytical and experimental investigations, the conclusions are highlighted as follows:

The results of damage localization indicated that the predicted and actual damage location were remarkably similar. Simultaneously, experiments have verified that the damage expansion was described appropriately by the proposed damage quantification approach. For the composite plate with a sensor spacing of 100 mm, the damage localization and quantification errors are within 11 mm and 2.2 mm, respectively. The damage detection method proposed in this paper has the potential to pinpoint the damage localization and quantification target.

Overall, the major limitation of this study is that the parameter was selected based on experimental data and experience in the process of numeralization of quantitative images, which means that different material architectures require different settings. Further research will focus on integrating the structural physical properties to parameters for adaptive parameter selection.

The proposed damage monitoring method is validated in composite plate-like structures. The research idea can be extended to other plate-like structures to achieve damage localization and quantification monitoring. However, for more complex structures, the continuous reflection and the interaction between Lamb waves make the interpretation of the Lamb wave propagation mechanism a challenge. Thus, it needs to be improved to achieve the goal of damage monitoring in complex structures.

## Figures and Tables

**Table 1:** Monitoring equipment technical indicators.

Technical Parameter	Value
Excitation Frequency Range	10–1000 kHz
Conversion Rates	48 MHz
Output Voltage Range	Min: ±10 V; Max: ±60 V
Memory	32,000 Samples
Sampling Rates	6, 12, 24, 48 MHz/s
Resolution	12-bit
ADC Range	±1 V
Gain Adjustment Range	10–40 dB, step: 1 dB

**Table 2:** Damage center prediction results and errors.

Actual Damage Location (mm)	Damage Diameter (mm)	Results of Using Hilbert to Extract TOF (mm)	Distance between Predicted and Actual Location (mm)	Relative Error (%)	Results of Using MPD to Extract TOF (mm)	Distance between Predicted and Actual Location (mm)	Relative Error (%)
(320, 245)	4	(342.9, 245.4)	22.9	5.08	(309.2, 242.7)	11.0	2.44
(320, 245)	8	(334.9, 241.5)	15.3	3.40	(313.2, 241.6)	7.6	1.69
(320, 245)	12	(336.6, 243.7)	16.6	3.69	(317.9, 252.5)	7.8	1.73
(127, 185)	4	(134.8, 179.5)	9.5	2.11	(134.4, 185.1)	7.4	1.64
(127, 185)	8	(135.1, 179.9)	9.6	2.13	(130.3, 191.9)	7.6	1.69
(127, 185)	12	(133.8, 176.2)	11.4	2.53	(116.1, 187.0)	11.0	2.44

**Table 3:** Damage quantification prediction results and errors.

Actual Damage Location (mm)	Damage Diameter (mm)	Actual Damage Size (mm^2^)	Predicted Damage Size (mm^2^)	Corresponding Predicted Damage Diameter(mm)	Absolute Error (mm^2^)
(320, 245)	4	12.56	30.04	6.18	17.48
(320, 245)	8	50.24	47.14	7.75	3.10
(320, 245)	12	113.04	95.69	11.04	17.35
(127, 185)	4	12.56	14.26	4.26	1.70
(127, 185)	8	50.24	45.57	7.61	4.67
(127, 185)	12	113.04	85.53	10.43	27.51

## Data Availability

The data presented in this study are available in the article.
